# Adverse Childhood Experiences and Subsequent Chronic Diseases Among Middle-aged or Older Adults in China and Associations With Demographic and Socioeconomic Characteristics

**DOI:** 10.1001/jamanetworkopen.2021.30143

**Published:** 2021-10-25

**Authors:** Li Lin, Harry Haoxiang Wang, Ciyong Lu, Weiqing Chen, Vivian Yawei Guo

**Affiliations:** 1 School of Public Health, Sun Yat-sen University, Guangzhou, China

## Abstract

**Importance:**

Associations between adverse childhood experiences (ACEs) and chronic diseases among middle-aged or older Chinese individuals have not been well documented. In addition, whether demographic and socioeconomic characteristics modify any such associations has been underexplored.

**Objectives:**

To examine associations between ACEs and subsequent chronic diseases and to assess whether age, sex, educational level, annual per capita household expenditure level, and childhood economic hardship modify these associations.

**Design, Setting, and Participants:**

This population-based cross-sectional study used data from the China Health and Retirement Longitudinal Study (CHARLS), a survey of residents aged 45 years or older in 28 provinces across China; specifically, the study used data from the CHARLS life history survey conducted from June 1 to December 31, 2014, and a CHARLS follow-up health survey conducted from July 1 to September 30, 2015. The study population included 11 972 respondents aged 45 years or older who had data on at least 1 of 14 specified chronic diseases and information on all 12 of the ACE indicators included in this study. Data analysis was performed from December 1 to 30, 2020.

**Exposures:**

Any of 12 ACEs (physical abuse, emotional neglect, household substance abuse, household mental illness, domestic violence, incarcerated household member, parental separation or divorce, unsafe neighborhood, bullying, parental death, sibling death, and parental disability), measured by indicators on a questionnaire. The number of ACEs per participant was summed and categorized into 1 of 5 cumulative-score groups: 0, 1, 2, 3, and 4 or more.

**Main Outcomes and Measures:**

Hypertension, dyslipidemia, diabetes, heart disease, stroke, chronic lung disease, asthma, liver disease, cancer, digestive disease, kidney disease, arthritis, psychiatric disease, and memory-related disease were defined by self-reported physician diagnoses or in combination with health assessment and medication data. Multimorbidity was defined as the presence of 2 or more of these 14 chronic diseases. Logistic regression models were used to assess associations of the 12 ACEs with the 14 chronic diseases and with multimorbidity. Modification of the associations by demographic and socioeconomic characteristics was assessed by stratified analyses and tests for interaction.

**Results:**

Of the 11 972 individuals included (mean [SD] age, 59.85 [9.56] years; 6181 [51.6%] were females), 80.9% had been exposed to at least 1 ACE and 18.0% reported exposure to 4 or more ACEs. Compared with those without ACE exposure, participants who experienced 4 or more ACEs had increased risks of dyslipidemia, chronic lung disease, asthma, liver disease, digestive disease, kidney disease, arthritis, psychiatric disease, memory-related disease, and multimorbidity. The estimated odds ratios (ORs) ranged from 1.27 (95% CI, 1.02-1.59) for dyslipidemia to 2.59 (95% CI, 2.16-3.11) for digestive disease. A dose-response association was also observed between the number of ACEs and the risk of most of the chronic diseases (excluding hypertension, diabetes, and cancer) (eg, chronic lung disease for ≥4 ACEs vs none: OR, 2.01; 95% CI, 1.59-2.55; *P* < .001 for trend) and of multimorbidity (for individuals among the overall study population with ≥4 ACEs vs none: OR, 2.03; 95% CI, 1.70-2.41; *P* < .001 for trend). The demographic or socioeconomic characteristics of age, sex, educational level, annual per capita household expenditure level, or childhood economic hardship were not shown to significantly modify the associations between ACEs and multimorbidity.

**Conclusions and Relevance:**

In this population-based, cross-sectional study of adults in China, exposure to ACEs was associated with higher risks of chronic diseases regardless of demographic and socioeconomic characteristics during childhood or adulthood. These findings suggest a need to prevent ACEs and a need for a universal life-course public health strategy to reduce potential adverse health outcomes later in life among individuals who experience them.

## Key Points

**Question:**

Are adverse childhood experiences (ACEs) associated with subsequent chronic diseases among middle-aged or older adults in China, and do demographic and socioeconomic characteristics modify these associations?

**Findings:**

In this cross-sectional study of 11 972 Chinese individuals aged 45 years or older, dose-response associations were found between the number of ACEs to which a participant was exposed and increased risks of various chronic diseases and multimorbidity. Age, sex, educational level, annual per capita household expenditure level, and childhood economic hardship did not significantly modify these associations.

**Meaning:**

Interventions implemented among individuals exposed to ACEs may help to reduce the potential burden of later-life chronic diseases.

## Teaser

This cross-sectional study uses data from the China Health and Retirement Longitudinal Study to examine associations between adverse childhood experiences and chronic diseases later in life among adults in China.

## Introduction

Adverse childhood experiences (ACEs), or childhood adversities, refer to a wide range of potentially stressful experiences that occur in childhood. The most widely used ACE scales, ie, the conventional ACEs, include 10 items covering abuse, neglect, and household challenges from the Centers for Disease Control and Prevention (CDC)–Kaiser Permanente ACE Study.^1,2^ However, it is generally accepted that conventional ACEs may not adequately reflect perceived childhood adversity in populations that are different from those in the CDC–Kaiser Permanente ACE Study.^3^ An additional set of expanded ACEs have been included to measure community-level stressors such as neighborhood or school bullying and unsafe communities.^4^ In addition, several new ACE indicators (eg, somatic illness or death of family members) have also been reported to be prevalent and should be considered in ACE-related studies.^5,6^

A substantial amount of literature has also included socioeconomic characteristics as an individual ACE component and explored its associations with deleterious consequences for health.^5,6,7^ In contrast, some studies have argued that socioeconomic characteristics should be examined as a separate construct different from ACEs because the association of ACEs with health outcomes might be mitigated in people with a high socioeconomic position.^8,9^ However, a prospective cohort study in the UK showed that there was no difference in the association of ACEs with educational attainment and health outcomes across different socioeconomic groups.^9^ Similar findings have also been reported by other studies regardless of income group, ethnicity, or socioeconomic position.^10,11,12^ Nevertheless, associations between ACEs, demographic and socioeconomic characteristics, and chronic diseases in later life have been underexplored. In addition, although the literature has shown associations between ACE exposure and mental and physical illness later in life,^7,13^ few studies have focused on such associations in developing countries such as China, where ACEs are more prevalent.^14,15^

In this study, we collected information on 12 ACE indicators among Chinese adults using data from the China Health and Retirement Longitudinal Study (CHARLS), a nationwide survey of representative residents aged 45 years or older. We investigated whether there were associations between ACEs and 14 noncommunicable chronic diseases as well as multimorbidity. Stratified analyses and tests for interaction were conducted to further evaluate whether associations were modified by participants’ age, sex, educational level, annual per capita household expenditure level, and childhood economic hardship.

## Methods

### Study Design and Population

This cross-sectional study was conducted using data from the CHARLS. The detailed study design and sampling methods have been reported previously.^16^ In brief, participants in CHARLS were randomly selected using a multistage probability sampling strategy. The baseline survey consisted of 17 708 participants from 450 villages or resident communities in 28 provinces across China. Respondents were followed up every 2 years and a small share of new participants was recruited in every survey. To date, 3 follow-up surveys have been conducted in 2013, 2015, and 2018. Information on childhood experiences was also collected in the 2014 life history survey among all living respondents in the 2011 and 2013 surveys. The current analysis used data from the 2014 life history survey, conducted from June 1 to December 31, 2014, and the 2015 follow-up survey, conducted from July 1 to September 30, 2015, when the latest health assessment information was available. Data analysis was performed from December 1 to 30, 2020. The CHARLS has received ethical approval from the Institutional Review Board of Peking University.^16^ Written informed consent was obtained from all participants. According to the London School of Economics and Political Science research ethics policy and procedures, the current study was not subject to ethical approval or informed consent because a secondary analysis was conducted using established data sets. This study followed the Strengthening the Reporting of Observational Studies in Epidemiology (STROBE) reporting guideline.

**Figure 1.**
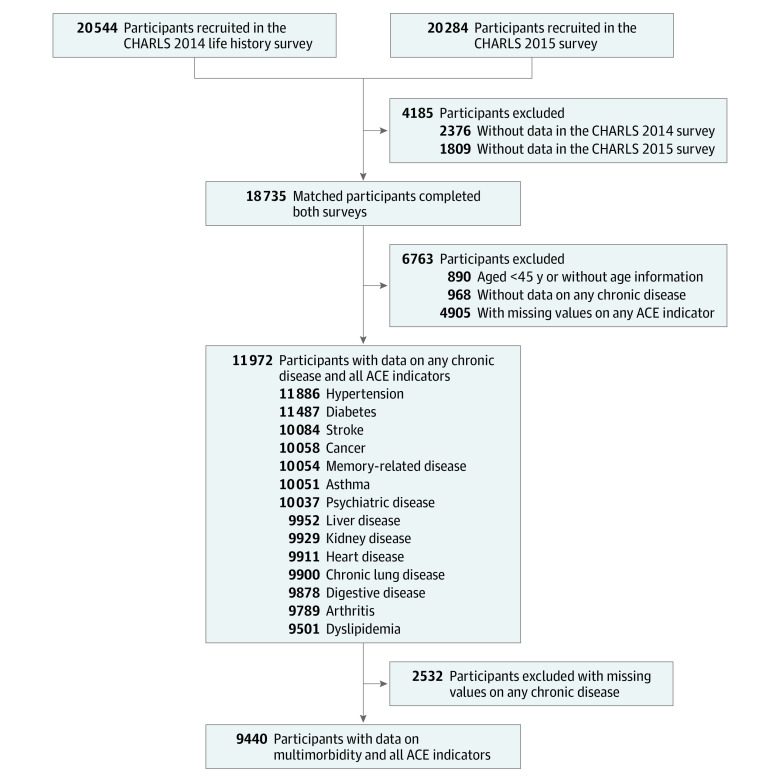
Flowchart of Study Participant Selection ACE indicates adverse childhood experience; CHARLS, China Health and Retirement Longitudinal Study.

A total of 20 544 individuals participated in the CHARLS 2014 life history survey and 20 284 participated in the 2015 follow-up survey (Figure 1). We successfully conducted 1:1 matching of 18 735 respondents who had completed both surveys. After exclusion of 890 participants without age information or who were younger than 45 years of age, 968 individuals without any data on chronic disease status, and 4905 individuals with missing information on any of the 12 included ACE components, 11 972 participants with information on at least 1 of the 14 chronic diseases included in this study and complete data on all 12 of the ACE components were included to evaluate associations between ACEs and chronic diseases. The number of participants with each of the included chronic diseases is shown in Figure 1. To assess associations between ACEs and multimorbidity, we further excluded 2532 participants without complete data on the 14 chronic diseases, leaving 9440 individuals in this statistical analysis.

### Definitions of Chronic Diseases

Each participant’s disease status (yes or no) for a total of 14 noncommunicable chronic diseases including hypertension, dyslipidemia, diabetes, heart disease, stroke, chronic lung disease, asthma, liver disease, cancer, digestive disease, kidney disease, arthritis, psychiatric disease, and memory-related disease was confirmed by the patient’s self-report of a physician’s diagnosis or in combination with health assessment and medication data in the 2015 CHARLS survey. Detailed definitions of these chronic diseases are provided in the eMethods in the Supplement. Multimorbidity was defined as the coexistence of 2 or more of these 14 chronic diseases in the same individual.

### Definition of Adverse Childhood Experiences

We extracted 12 ACEs from the CHARLS data set, including 7 conventional ACEs (physical abuse, emotional neglect, household substance abuse, household mental illness, domestic violence, incarcerated household member, and parental separation or divorce),^1,2^ 2 ACEs from the expanded set (unsafe neighborhood and bullying),^4^ and 3 of the new ACEs that have been reported previously (parental death, sibling death, and parental disability).^5,6^ The detailed questionnaire items and definitions of each ACE indicator are available in eTable 1 in the Supplement. Responses to each item were dichotomized and summed to generate a cumulative ACE score for each participant, ranging from 0 to 12. We further categorized participants into 5 groups based on the cumulative ACE scores: 0, 1, 2, 3, and 4 or higher.

### Other Measures

Data on age, sex, ethnicity, marital status, educational level, rural or urban residence, smoking and drinking status, annual per capita household expenditure level, and childhood economic hardship were collected through face-to-face interviews. The annual per capita household expenditure was used to reflect current economic status, because previous research reported that it was a better indicator of living standards than household income, especially in rural areas.^17^ Body mass index (BMI) was calculated as weight in kilograms divided by height in meters squared. Participants with a BMI of 28 or greater were defined as having obesity based on the recommended standard for Chinese adults.^18^ More details about these variables are provided in the eMethods in the Supplement.

### Statistical Analysis

For comparisons of characteristics across different ACE groups, analysis of variance was used for continuous variables and χ^2^ tests were applied for categorical variables. To assess trends in characteristics across different ACE groups, polynomial comparisons were used to analyze variance in trends for continuous data and the Mantel-Haenszel statistic was used for categorical data.

Logistic regression models were established to assess the associations between ACE groups and each individual chronic disease. Model 1 was a crude model. Model 2 was adjusted for age, sex, ethnicity, marital status, educational level, rural or urban residence, smoking and drinking status, annual per capita household expenditure level, and childhood economic hardship. Trend tests were performed to assess whether a dose-response association was present. A sensitivity analysis including only the 7 conventional ACEs was further conducted. We then assessed associations between ACE groups and multimorbidity using logistic regression models adjusted for the same covariates listed in model 2. Odds ratios (ORs) and 95% CIs are reported for all regression models.

To identify potential modifying variables, stratified analyses were conducted for age group (45-59 years and ≥60 years), sex (females or males), educational level completed (primary school or below and middle school or above), annual per capita household expenditure level (in tertiles), and childhood economic hardship (yes or no). To adequately address missing data, we also reanalyzed the associations of ACE groups with chronic disease and multimorbidity using 50 imputed data sets with the multiple imputation method by chained equations.

All statistical analyses were conducted using Stata, version 15.0 (StataCorp LLC). All *P* values were 2-sided, and significance was set at *P* < .05.

## Results

Of the 11 972 participants included, 6181 (51.6%) were females, 5790 (48.4%) were males, and the mean (SD) age was 59.85 (9.56) years. The prevalence of each individual ACE component ranged from 0.3% (incarcerated household members) to 34.5% (emotional neglect) (eTable 1 in the Supplement). Overall, 80.9% of the participants had been exposed to at least 1 of the 12 ACEs and 18.0% had experienced 4 or more ACEs. Of the participants with ACEs, 14.2% had experienced ACEs from only the expanded or new set (eFigure 1 in the Supplement). In general, compared with participants without any ACE exposure, those who reported 4 or more ACEs were more likely to be older, less educated, unmarried, and residents of a rural area (Table 1). We observed that an increase in the number of ACEs was associated with an increasing trend in the prevalence of multimorbidity and chronic diseases with the exception of hypertension, dyslipidemia, diabetes, and cancer.

**Table 1.** Characteristics of Participants by Number of ACEs

Characteristics^a^	ACEs, No. (N = 11 972)					*P* value	*P* value for trend
	0 (n = 2292)	1 (n = 3220)	2 (n = 2605)	3 (n = 1703)	≥4 (n = 2152)		
Age, y	58.74 (9.12)	59.53 (9.45)	59.86 (9.65)	60.58 (9.79)	60.92 (9.74)	<.001	<.001
Sex							
Male	967 (42.2)	1514 (47.0)	1307 (50.2)	912 (53.6)	1090 (50.7)	<.001	<.001
Female	1325 (57.8)	1706 (53.0)	1297 (49.8)	791 (46.4)	1062 (49.3)		
Ethnicity							
Han ethnicity	2094 (91.6)	2949 (91.9)	2393 (92.1)	1578 (92.8)	1987 (92.5)	.64	.15
Ethnic minority populations	191 (8.4)	260 (8.1)	205 (7.9)	122 (7.2)	161 (7.5)		
Marital status							
Married	2064 (90.1)	2835 (88.1)	2278 (87.6)	1497 (87.9)	1831 (85.1)	<.001	<.001
Unmarried	227 (9.9)	383 (11.9)	323 (12.4)	206 (12.1)	320 (14.9)		
Educational level completed							
Primary school or below	1199 (57.5)	1841 (62.0)	1534 (64.0)	1075 (68.0)	1484 (74.6)	<.001	<.001
Middle school or above	887 (42.5)	1129 (38.0)	864 (36.0)	505 (32.0)	506 (25.4)		
Area of residence							
Rural	1382 (60.3)	1926 (59.8)	1609 (61.8)	1076 (63.2)	1439 (66.9)	<.001	<.001
Urban	910 (39.7)	1294 (40.2)	996 (38.2)	627 (36.8)	713 (33.1)		
Childhood economic hardship							
Yes	538 (23.5)	956 (29.8)	972 (37.3)	790 (46.4)	1276 (59.5)	<.001	<.001
No	1751 (76.5)	2257 (70.2)	1632 (62.7)	913 (53.6)	870 (40.5)		
Annual per capita household expenditure level							
Tertile 1	585 (33.9)	820 (33.7)	667 (34.2)	455 (36.1)	565 (36.1)	.64	.05
Tertile 2	585 (33.9)	841 (34.7)	657 (33.6)	414 (32.9)	538 (34.4)		
Tertile 3	554 (32.2)	769 (31.6)	628 (32.2)	391 (31.0)	462 (29.5)		
Smoking status							
Never smoker	1410 (62.8)	1834 (58.6)	1376 (54.4)	875 (53.0)	1132 (54.2)	<.001	<.001
Former smoker	288 (12.8)	394 (12.6)	373 (14.8)	245 (14.8)	289 (13.8)		
Current smoker	547 (24.4)	901 (28.8)	779 (30.8)	530 (32.1)	667 (31.9)		
Drinking status							
Never drinker	1368 (59.8)	1779 (55.4)	1305 (50.3)	846 (49.7)	1023 (47.6)	<.001	<.001
Former drinker	218 (9.5)	338 (10.5)	290 (11.2)	191 (11.2)	304 (14.1)		
Current drinker	703 (30.7)	1097 (34.1)	1002 (38.6)	664 (39.0)	822 (38.3)		
BMI	24.19 (3.84)	24.06 (3.71)	24.07 (3.74)	23.89 (3.84)	23.62 (3.83)	<.001	<.001
Obesity^b^							
Yes	302 (15.5)	387 (14.1)	304 (13.7)	171 (11.8)	226 (12.1)	.006	<.001
No	1652 (84.5)	2360 (85.9)	1923 (86.3)	1284 (88.2)	1647 (87.9)		
Chronic disease							
Hypertension	875 (38.5)	1276 (39.8)	1074 (41.5)	671 (39.8)	820 (38.4)	.16	.96
Dyslipidemia	292 (15.9)	410 (15.9)	357 (17.2)	225 (16.7)	278 (16.7)	.77	.37
Diabetes	355 (16.1)	479 (15.6)	419 (16.7)	264 (16.1)	356 (17.3)	.51	.19
Heart disease	313 (16.4)	423 (15.8)	373 (17.2)	267 (18.8)	308 (17.7)	.14	.047
Stroke	47 (2.4)	56 (2.1)	70 (3.2)	59 (4.1)	70 (3.9)	<.001	<.001
Chronic lung disease	177 (9.3)	258 (9.6)	278 (12.7)	231 (16.4)	335 (19.3)	<.001	<.001
Asthma	71 (3.7)	102 (3.8)	113 (5.1)	95 (6.7)	138 (7.8)	<.001	<.001
Liver disease	92 (4.8)	122 (4.5)	139 (6.4)	84 (5.9)	129 (7.4)	<.001	<.001
Cancer	27 (1.4)	39 (1.4)	38 (1.7)	16 (1.1)	31 (1.7)	.54	.59
Digestive disease	372 (19.6)	654 (24.5)	607 (28.0)	447 (32.0)	667 (38.3)	<.001	<.001
Kidney disease	122 (6.4)	215 (8.0)	193 (8.9)	142 (10.0)	217 (12.5)	<.001	<.001
Arthritis	587 (31.1)	889 (33.6)	866 (40.5)	626 (44.7)	869 (5.6)	<.001	<.001
Psychiatric disease	27 (1.4)	31 (1.1)	36 (1.6)	26 (1.8)	56 (3.2)	<.001	<.001
Memory-related disease	34 (1.8)	54 (2.0)	59 (2.7)	37 (2.6)	64 (3.6)	.002	<.001
Multimorbidity	915 (50.8)	1352 (53.2)	1272 (61.0)	859 (64.4)	1163 (69.3)	<.001	<.001

Abbreviations: ACE, adverse childhood experience; BMI, body mass index (calculated as weight in kilograms divided by height in meters squared).

^a^ Continuous data are reported as the mean (SD), and categorical data are reported as the number and percentage of participants.

^b^ Defined as a BMI of 28 or greater.

In the main analysis of the associations between ACE groups and individual chronic diseases, we found that experiencing 4 or more ACEs was associated with a higher risk of dyslipidemia, chronic lung disease, asthma, liver disease, digestive disease, kidney disease, arthritis, psychiatric disease, and memory-related disease compared with no ACE exposure (Table 2). The adjusted ORs ranged from 1.27 (95% CI, 1.02-1.59) for dyslipidemia to 2.59 (95% CI, 2.16-3.11) for digestive disease. We also observed a significant dose-response association between cumulative ACE scores and each of the chronic diseases with the exception of hypertension, diabetes, and cancer. Similar to the findings of the main statistical analysis, sensitivity analyses revealed associations between conventional ACEs and chronic diseases with the exception of liver disease (eTable 2 in the Supplement).

**Table 2.** Association Between the Number of ACEs and Subsequent Chronic Diseases in Adulthood

Chronic disease	OR (95% CI) by No. of ACEs					*P* value for trend
	0	1	2	3	≥4	
**Model 1^a^**						
Hypertension	1 [Reference]^b^	1.06 (0.95-1.18)	1.14 (1.01-1.28)	1.06 (0.93-1.20)	1.00 (0.88-1.13)	.98
Dyslipidemia	1 [Reference]	1.00 (0.85-1.18)	1.09 (0.92-1.29)	1.06 (0.88-1.28)	1.06 (0.88-1.26)	.37
Diabetes	1 [Reference]	0.97 (0.83-1.12)	1.05 (0.90-1.23)	1.00 (0.84-1.19)	1.10 (0.94-1.29)	.18
Heart disease	1 [Reference]	0.96 (0.81-1.12)	1.05 (0.89-1.24)	1.18 (0.98-1.41)	1.09 (0.92-1.30)	.047
Stroke	1 [Reference]	0.84 (0.57-1.25)	1.31 (0.90-1.90)	1.72 (1.16-2.54)	1.64 (1.12-2.38)	<.001
Chronic lung disease	1 [Reference]	1.03 (0.85-1.26)	1.42 (1.16-1.73)	1.91 (1.55-2.36)	2.32 (1.91-2.82)	<.001
Asthma	1 [Reference]	1.02 (0.75-1.39)	1.41 (1.04-1.91)	1.87 (1.36-2.56**)**	2.20 (1.64-2.95)	<.001
Liver disease	1 [Reference]	0.94 (0.72-1.24)	1.35 (1.03-1.77)	1.25 (0.92-1.69)	1.58 (1.20-2.09)	<.001
Cancer	1 [Reference]	1.03 (0.63-1.69)	1.24 (0.75-2.04)	0.80 (0.43-1.49)	1.26 (0.75-2.11)	.59
Digestive disease	1 [Reference]	1.33 (1.15-1.54)	1.60 (1.38-1.85)	1.93 (1.64-2.26)	2.56 (2.20-2.97)	<.001
Kidney disease	1 [Reference]	1.27 (1.01-1.60)	1.42 (1.12-1.80)	1.62 (1.26-2.09)	2.10 (1.66-2.65)	<.001
Arthritis	1 [Reference]	1.12 (0.99-1.27)	1.51 (1.32-1.72)	1.80 (1.56-2.07)	2.26 (1.98-2.59)	<.001
Psychiatric disease	1 [Reference]	0.82 (0.49-1.37)	1.17 (0.71-1.94)	1.31 (0.76-2.25)	2.31 (1.45-3.68)	<.001
Memory-related disease	1 [Reference]	1.13 (0.73-1.74)	1.53 (1.00-2.34)	1.48 (0.92-2.37)	2.08 (1.37-3.17)	<.001
**Model 2^c^**						
Hypertension	1 [Reference]	1.02 (0.89-1.18)	1.09 (0.95-1.26)	0.98 (0.83-1.15)	0.85 (0.73-1.00)	.051
Dyslipidemia	1 [Reference]	1.04 (0.85-1.26)	1.16 (0.95-1.43)	1.17 (0.93-1.48)	1.27 (1.02-1.59)	.01
Diabetes	1 [Reference]	1.02 (0.85-1.23)	1.16 (0.96-1.40)	1.01 (0.81-1.26)	1.16 (0.94-1.43)	.17
Heart disease	1 [Reference]	0.96 (0.79-1.16)	1.05 (0.86-1.28)	1.22 (0.98-1.52)	1.18 (0.95-1.46)	.02
Stroke	1 [Reference]	0.88 (0.54-1.42)	1.25 (0.78-2.00)	1.53 (0.93-2.51)	1.55 (0.97-2.50)	.006
Chronic lung disease	1 [Reference]	0.98 (0.78-1.24)	1.33 (1.05-1.68)	1.70 (1.32-2.18)	2.01 (1.59-2.55)	<.001
Asthma	1 [Reference]	0.95 (0.66-1.38)	1.36 (0.95-1.96)	1.73 (1.18-2.53)	2.23 (1.56-3.19)	<.001
Liver disease	1 [Reference]	0.84 (0.61-1.15)	1.23 (0.91-1.68)	1.15 (0.81-1.63)	1.45 (1.05-2.01)	.002
Cancer	1 [Reference]	0.99 (0.56-1.75)	1.22 (0.68-2.19)	0.79 (0.37-1.66)	1.09 (0.57-2.09)	.92
Digestive disease	1 [Reference]	1.40 (1.18-1.65)	1.63 (1.37-1.95)	2.04 (1.68-2.47)	2.59 (2.16-3.11)	<.001
Kidney disease	1 [Reference]	1.20 (0.92-1.56)	1.22 (0.92-1.61)	1.31 (0.97-1.78)	1.98 (1.50-2.62)	<.001
Arthritis	1 [Reference]	1.06 (0.91-1.23)	1.42 (1.22-1.66)	1.62 (1.37-1.93)	1.97 (1.67-2.33)	<.001
Psychiatric disease	1 [Reference]	0.73 (0.39-1.34)	1.27 (0.72-2.24)	1.35 (0.72-2.53)	2.28 (1.31-3.94)	<.001
Memory-related disease	1 [Reference]	1.07 (0.63-1.83)	1.34 (0.78-2.27)	1.46 (0.82-2.59)	1.99 (1.18-3.37)	.003

Abbreviations: ACE, Adverse Childhood Experience; OR, odds ratio.

^a^ Model 1 was the crude model.

^b^ Reference: No ACE exposure.

^c^ Model 2 was adjusted for age, sex, ethnicity, marital status, educational level, rural or urban residence, smoking and drinking status, annual per capita household expenditure level, and childhood economic hardship.

**Figure 2.**
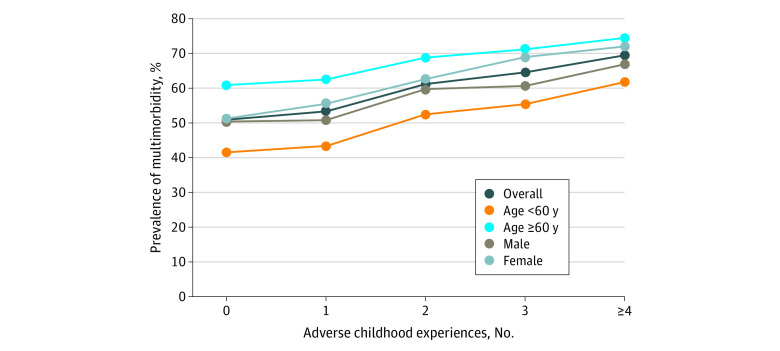
Prevalence of Multimorbidity by Number of Adverse Childhood Experiences in the Overall Study Population Stratified by Age and Sex

Of the 9440 participants with status data for all 14 chronic diseases, a total of 5561 individuals (58.9%) had multimorbidity. Increases in cumulative ACE scores were associated with increased prevalence of multimorbidity in the overall sample as well as in different age and sex groups (Figure 2). We found that 11 of the 12 ACEs (except incarcerated household members) were associated with increased odds of having multimorbidity, although some were not statistically significant (eFigure 2 in the Supplement). In the fully adjusted model, when considering outcomes associated with higher cumulative ACE scores, we found that participants with 4 or more ACEs had an approximately 2-fold increased risk of multimorbidity (OR, 2.03; 95% CI, 1.70-2.41) compared with those with no ACEs (Table 3). Similar outcomes were also found in different subgroups with significant dose-response associations. However, we did not find that age, sex, educational level, annual per capita household expenditure level, or childhood economic hardship significantly modified the associations between ACE groups and multimorbidity.

**Table 3.** Association Between the Number of ACEs and Multimorbidity in the Overall Study Population and Subgroups

Characteristic	OR (95% CI) by No. of ACEs^a^					*P* value for trend	*P* value for interaction
	0	1	2	3	≥4		
Overall study population	1 [Reference]^b^	1.09 (0.94-1.26)	1.39 (1.19-1.62)	1.71 (1.43-2.05)	2.03 (1.70-2.41)	<.001	
Subgroup							
Age, y							
<60	1 [Reference]	1.10 (0.90-1.35)	1.43 (1.16-1.76)	1.79 (1.39-2.30)	2.17 (1.70-2.77)	<.001	.15
≥60	1 [Reference]	1.07 (0.86-1.32)	1.36 (1.09-1.71)	1.65 (1.28-2.12)	1.93 (1.51-2.47)	<.001	
Sex							
Male	1 [Reference]	1.04 (0.83-1.30)	1.42 (1.13-1.79)	1.48 (1.15-1.91)	2.04 (1.58-2.64)	<.001	.85
Female	1 [Reference]	1.14 (0.93-1.38)	1.36 (1.10-1.69)	2.06 (1.59-2.67)	2.03 (1.60-2.57)	<.001	
Childhood economic hardship							
Yes	1 [Reference]	1.10 (0.83-1.46)	1.47 (1.11-1.96)	1.82 (1.34-2.47)	2.02 (1.52-2.67)	<.001	.93
No	1 [Reference]	1.09 (0.92-1.29)	1.35 (1.12-1.62)	1.66 (1.32-2.08)	2.11 (1.67-2.67)	<.001	
Educational level completed							
Primary school or below	1 [Reference]	1.14 (0.94-1.38)	1.49 (1.21-1.82)	1.82 (1.45-2.28)	2.25 (1.81-2.80)	<.001	.43
Middle school or above	1 [Reference]	1.02 (0.81-1.27)	1.24 (0.97-1.58)	1.58 (1.18-2.12)	1.63 (1.21-2.19)	<.001	
Annual per capita household expenditure level							
Tertile 1	1 [Reference]	1.25 (0.98-1.61)	1.33 (1.02-1.72)	1.66 (1.24-2.24)	2.42 (1.81-3.24)	<.001	.94
Tertile 2	1 [Reference]	1.08 (0.84-1.38)	1.62 (1.24-2.12)	1.91 (1.40-2.61)	1.91 (1.43-2.57)	<.001	
Tertile 3	1 [Reference]	0.94 (0.72-1.23)	1.24 (0.93-1.63)	1.65 (1.19-2.29)	1.79 (1.29-2.48)	<.001	

Abbreviations: ACE, adverse childhood experience; OR, odds ratio.

^a^ The model was adjusted for age, sex, ethnicity, marital status, educational level, rural or urban residence, smoking and drinking status, annual per capita household expenditure level, and childhood economic hardship, except for the stratified variables in each subgroup.

^b^ Reference: No ACE exposure.

When incomplete data were imputed with the multiple imputation method, the results were consistent with the study’s main findings (eTable 3 and eTable 4 in the Supplement).

## Discussion

In this cross-sectional study, exposure to ACEs was associated with multiple chronic diseases and multimorbidity among adults in China. A significant dose-response association between the number of ACEs to which individuals were exposed and the prevalence of chronic diseases was also observed, with the exception of hypertension, diabetes, and cancer. Stratified analyses revealed patterns similar to those of the main findings. The findings did not indicate that age, sex, educational level, annual per capita household expenditure level, or childhood economic hardship modified the associations between ACEs and multimorbidity.

Although the 10-item conventional ACEs from the CDC–Kaiser Permanente ACE Study are widely used across research literature in different populations, the scales were generated based on a sample of mostly White and educated individuals.^1,2^ As such, the perceived adversities and types of adversities might be different across different populations. Several studies and reviews have pointed out the need for inclusion of additional ACE items, but there is still ongoing debate about which indicators should be included.^3,4^ In this study, if only conventional ACEs were counted, ACE exposure would have been underreported in 14.2% of the middle-aged or older Chinese adults included. Furthermore, this study’s results suggest that the burden and magnitude of some of the additional ACEs not included in the CDC–Kaiser Permanente ACE Study were salient and should not be ignored. These results indicate that efforts may still be needed to explore culturally relevant ACE scales.

This study’s findings of associations between ACEs and chronic diseases later in life were consistent with those of previous studies.^7,19^ Nevertheless, the exact underlying mechanisms are unclear. One possible explanation is that prolonged stress caused by ACEs may lead to chronic activation of the hypothalamic-pituitary-adrenal axis and subsequently to increased allostatic load and disruption of the regulatory systems of the body, including the neuroendocrine, immune, metabolic, autonomic nervous, and cardiovascular systems.^20,21^ Previous studies have also suggested that ACEs may be associated with increased cortisol levels and chronic inflammation,^22^ which in turn may be associated with increased risk of several chronic diseases.^23^ In addition, studies have shown an association of ACEs with DNA methylation in key genes and with telomere length shortening, potentially resulting in a greater risk of developing age-related diseases.^24,25,26^

Another possible explanation is associated with behavioral problems after exposure to ACEs. Children who experience ACEs may have impaired development of the brain region that is associated with coping, planning, learning, self-regulation, and management.^27^ Therefore, they may be more likely to have behavioral problems in adulthood, such as heavy smoking, alcohol abuse, and sleeping disorders,^1,7^ which are well-established factors associated with the risk of physical and mental illness.^28^ This study’s findings also showed that the prevalence of ever smoking and ever drinking was positively associated with the number of ACEs to which an individual had been exposed.

The prevalence of obesity in this study’s sample was the highest among individuals with no exposure to ACEs (15.5%) and the lowest among those exposed to 4 or more ACEs (12.1%), which was contrary to the findings of existing studies.^7,29^ One reason for the discrepancy might be the differential pattern of obesity rates in different socioeconomic groups between developed and developing countries. Individuals with more ACE exposure have been found to be more likely to have low socioeconomic status, which in developed countries has been associated with a diet that consists of more energy-dense foods, whereas people of higher socioeconomic status can afford and demand a healthier diet (eg, low-calorie food) and exercise^30^; therefore, ACE exposure has been associated with greater risk of obesity in developed countries. In contrast, in developing countries, people in socioeconomically disadvantaged groups tend to have limited food resources and nutrition intake,^31^ whereas those from a higher socioeconomic class in developing countries may be able to afford and have access to surplus or excess food, and subsequently, they may have a higher prevalence of obesity.^31,32^ As such, we speculate that in this study, the nonsignificant association of ACE exposure with hypertension and diabetes might be owed to the association of a greater number of ACEs with lower obesity rates in the study’s Chinese population, which may have attenuated the potential detrimental health outcomes associated with long-term stress from ACEs. However, future longitudinal studies should further investigate the associations between ACEs and metabolic diseases in developing countries as well as the role obesity may have in the associations.

One major finding of this study was that demographic and socioeconomic characteristics were not significant modifiers of the associations between ACEs and multimorbidity; this observation was consistent with the findings of several previous studies investigating the associations between ACEs and outcomes according to ethnicity or socioeconomic factors.^9,10,11,12^ In contrast, a recent study in Japan reported that community-level social capital in later life modified the association between ACE exposure and incident dementia.^33^ Two studies have also shown that social support may mitigate the association between ACEs and detrimental outcomes.^34,35^ The findings of these studies suggest that social resources in later life, rather than individual-level demographic or socioeconomic characteristics, might be key modifiers to buffer deleterious outcomes of ACEs on health in later life. However, further studies are needed.

### Strengths and Limitations

A strength of this study was the large study sample used to explore the associations between ACEs and 14 different chronic diseases. In particular, we defined diabetes and hypertension using self-reported physician diagnoses and health examination data, which allowed us to identify undiagnosed cases. Furthermore, we conceptualized ACEs both as a cumulative score based on the total number of ACEs experienced and as individual components to show associations of different ACEs with health outcomes. We also explored whether several demographic and socioeconomic factors modified the association between ACEs and health outcomes, something that has been underexamined.

This study also has limitations. First, because a large proportion of participants was excluded from the data analysis owing to missing data, the representativeness of the study’s findings should be interpreted with caution. However, significant associations of ACEs with various chronic diseases and multimorbidity remained after multiple imputation of missing variables, suggesting the robustness of the study’s findings. Second, data on ACE indicators were collected retrospectively and were therefore subject to recall bias, especially for less objective events such as neglect. This was also suggested by the study’s finding of a lower multimorbidity OR for the variable of emotional neglect (eFigure 2 in the Supplement). Despite the risk of recall bias, previous research has reported the reliability of retrospective measures, which cannot be simply replaced by prospective measures.^36^ Nevertheless, further studies are needed to evaluate whether prospective and retrospective ACEs have different associations with chronic diseases.^36^ Third, we did not consider the frequency, intensity, and chronicity of ACEs, all of which have been found to be associated with poor health outcomes.^37^ In addition, this study assumed that the risk of each ACE component was equal, and the cumulative ACEs score was used in the statistical analyses. Although previous studies have reported that the pattern of associations between unweighted and weighted ACEs scores and inflammation and adiposity was similar, a weighted scoring method might enhance measurement precision.^38,39^ However, we were unable to assess this owing to data unavailability. Fourth, even though a wide range of ACE indicators were included in this study’s data analysis, some well-established ACE indicators such as sexual abuse and living in foster care^1,4^ were not included because the data were not available.

## Conclusions

The findings of this population-based cross-sectional study showed that a dose-response association existed between ACEs and increased risks of multiple chronic diseases and multimorbidity among middle-aged or older individuals in China. Age, sex, educational level, annual per capita household expenditure level, and childhood economic hardship did not significantly modify the associations. These findings suggest a need to prevent ACEs and a need for a life-course public health strategy to reduce potential associated risks of adverse health outcomes. Furthermore, emphasizing universal interventions that target individuals with ACE exposure may minimize the burden of associated chronic diseases later in life.

## Notes

Supplement. **eMethods.** Supplemental Methods **eTable 1.** Questionnaire Items and Prevalence of Each ACE Indicator **eTable 2.** Association Between the Number of Conventional ACEs and Subsequent Chronic Diseases and Multimorbidity in Adulthood **eTable 3.** Association Between the Number of ACEs and Subsequent Chronic Diseases, With Imputed Data Sets **eTable 4.** Association Between the Number of ACEs and Multimorbidity in the Overall Study Population and Subgroups, With Imputed Data Sets **eFigure 1.** Overlapping Exposure to Conventional, Expanded, and New ACEs **eFigure 2.** Association Between Individual ACE Indicator and Multimorbidity
eReferences
