# Errors in Figure

**DOI:** 10.1001/jamanetworkopen.2022.20614

**Published:** 2022-06-16

In the Original Investigation titled “Adverse Childhood Experiences and Subsequent Chronic Diseases Among Middle-aged or Older Adults in China and Associations With Demographic and Socioeconomic Characteristics,”^1^ published October 25, 2021, there were errors in the numbers of participants excluded and those without data in the CHARLS 2014 survey that appeared in the second-row box of Figure 1’s flow diagram. These should have been given as 3358 participants excluded and 1549 without data in the CHARLS 2014 survey. This article has been corrected.^1^
