# Supplementary material for: Adverse Childhood Experiences and Subsequent Chronic Diseases Among Middle-aged or Older Adults in China and Associations With Demographic and Socioeconomic Characteristics
Source: JAMA Netw Open. 2021 Oct 25;4(10):e2130143. doi: 10.1001/jamanetworkopen.2021.30143 (PMC8546496; doi:10.1001/jamanetworkopen.2021.30143)
Supplement: Supplement. — eMethods. Supplemental Methods eTable 1. Questionnaire Items and Prevalence of Each ACE Indicator eTable 2. Association Between the Number of Conventional ACEs and Subsequent Chronic Diseases and Multimorbidity in Adulthood eTable 3. Association Between the Number of ACEs and Subsequent Chronic Diseases, With Imputed Data Sets eTable 4. Association Between the Number of ACEs and Multimorbidity in the Overall Study Population and Subgroups, With Imputed Data Sets eFigure 1. Overlapping Exposure to Conventional, Expanded, and New ACEs eFigure 2. Association Between Individual ACE Indicator and Multimorbidity eReferences [file jamanetwopen-e2130143-s001.pdf]

Supplementary Online Content

Lin L, Wang HH, Lu C, Chen W, Guo VY. Adverse childhood experiences and subsequent chronic diseases among middle-aged or older adults in China and associations with demographic and socioeconomic characteristics. *JAMA Netw Open*. 2021;4(10):e2130143. doi:10.1001/jamanetworkopen.2021.30143

eMethods. Supplemental Methods

eTable 1. Questionnaire Items and Prevalence of Each ACE Indicator

eTable 2. Association Between the Number of Conventional ACEs and Subsequent Chronic Diseases and Multimorbidity in Adulthood

eTable 3. Association Between the Number of ACEs and Subsequent Chronic Diseases, With Imputed Data Sets

eTable 4. Association Between the Number of ACEs and Multimorbidity in the Overall Study Population and Subgroups, With Imputed Data Sets

eFigure 1. Overlapping Exposure to Conventional, Expanded, and New ACEs

eFigure 2. Association Between Individual ACE Indicator and Multimorbidity

eReferences

This supplementary material has been provided by the authors to give readers additional information about their work.

© 2021 Lin L et al. *JAMA Network Open*.

eMethods. Supplemental Methods

Definition of Chronic Diseases

The primary outcomes in this study were 14 noncommunicable chronic diseases for which data were collected in the 2015 CHARLS follow-up survey. Dyslipidemia, heart disease, stroke, chronic lung disease, asthma, liver disease, cancer, digestive disease, kidney disease, arthritis, psychiatric disease, and memory-related disease (including Alzheimer's disease, Parkinson's disease, and cerebral atrophy) were defined based on self-report of a physician's diagnosis. Blood pressure was measured three times with at least 45-second intervals using a digital sphygmomanometer (Omron ™ HEM-7200 Monitor, Co., LTD., Dalian, China).[1] Hypertension was defined as self-report of physician-diagnosed hypertension, and/or mean systolic blood pressure (SBP) ≥140 mmHg, and/or mean diastolic blood pressure (DBP) ≥90 mmHg, and/or on anti-hypertensive drugs.[2] Venous blood samples were also collected from each participant to obtain levels of plasma glucose and glycated hemoglobin (HbA1c). Plasma glucose and HbA1c levels were measured by the hexokinase method and high-performance liquid chromatography method, respectively. According to the American Diabetes Association criteria, a participant was defined as having diabetes if any of the following criteria was met: (1) fasting plasma glucose ≥7.0 mmol/L; (2) random plasma glucose ≥11.1 mmol/L; (3) HbA1c ≥6.5%; (4) self-reported diabetes diagnosed by a physician; and (5) on glucose-lower drugs/insulin treatment.[3]

The secondary outcome was multimorbidity, which was defined as the coexistence of two or more of these 14 chronic diseases in the same individual.[4] Participants without any chronic disease or with only one chronic disease were categorized into the non-multimorbidity group.

Other measures

Marital status was categorized into currently married/cohabitated and unmarried. The latter included never married, divorced, and widowed. Residence type was divided into urban or rural areas according to the classification of the villages or neighborhood communities by the National Bureau of Statistics of China.[5] Educational level completed was divided into two groups, i.e., primary school or below and middle school or above. Ethnicity was categorized into Han ethnicity and other ethnic minorities, as the Han ethnicity is the most populous ethnic group in China. Smoking and drinking status were categorized into never, former and current users based on self-report.

Previous research has shown that household consumption expenditure was a better indicator of living standards than household income, especially in rural areas.[6] Therefore, we used annual per capita household consumption expenditure level as an indicator of adulthood economic status. In the current study, household consumption expenditures (excluding medical expenditures) consisted of a wide range of expenditures, including household spending on food and eating out in the past week, communication and transportation expenditures in the past month, and cost on clothing and durable goods in the past year. The sum of above-mentioned expenditures over the past 12 months in the household was used to calculate the annual per capita household expenditure. Based on these data, we categorized participants into three levels by tertiles of the annual per capita household expenditure (tertile 1 [the lowest] to tertile 3 [the highest]).

Childhood economic hardship was ascertained by the question "When you were a child before the age of 17 years, compared to the average family in the same community/village at that time, how was your family's financial situation?" If the participant chose "a lot worse" or "somewhat worse", he/she was encoded as having economic hardship during childhood. In contrast, if "a lot better", "somewhat better" or "same as them" was selected, the participants were grouped as without economic hardship during their childhood.

© 2021 Lin L et al. *JAMA Network Open*.

eTable 1. Questionnaire Items and Prevalence of Each ACE Indicator.

Types of ACE	Domain	Questionnaire Items	Prevalence (%)
Conventional ACEs	Physical abuse	When you were growing up, did your female/male guardian ever hit you? (often[a], sometimes[a], rarely, or never)	30.8%
	Emotional neglect	How much love and affection did your female guardian give you while you were growing up? (often, sometimes, rarely[a], or never[a])	34.5%
		How much effort did your female guardian put into watching over you? (a lot, some, a little[a], or not at all[a])	
	Household substance abuse	During the years you were growing up, did your female/male guardian ever have alcoholism or drug? (yes[a] or no)	8.4%
	Household mental illness	Did your female/male guardian have abnormality of mind when you were young? (yes[a] or no)	14.8%
		During the years you were growing up, had your female/male guardian often showed continued signs of sadness or depression? (during all[a], most[a], some, or only a little of the childhood)	
	Domestic violence	Have your father/mother ever beat up your mother/father? (often[a], sometimes[a], not very often, or never)	22.7%
	Incarcerated household member	During the years you were growing up, have your female/male guardian ever been arrested or sent to prison? (yes[a] or no)	0.3%
	Parental separation or divorce	Were your biological parents divorced (including long separation due to emotional problems) before you were 17 years? (yes[a] or no)	0.5%
Expanded ACEs	Unsafe neighborhood	Was it safe being out alone at night in the neighborhood where you lived as a child? (very safe, somewhat safe, not very safe[a], or not safe at all[a])	9.1%
	Bullying	When you were a child, how often were you picked on or bullied by kids in your neighborhood? (often[a], sometimes[a], not very often, or never)	17.3%
		When you were a child, how often were you picked on or bullied by kids in your school? (often[a], sometimes[a], not very often, or never)	
New ACEs	Parental death[b]	Either of the parents was dead before participant was 17 years. (yes[a] or no)	17.6%
	Sibling death[c]	Any of the siblings was dead before participant was 17 years. (yes[a] or no)	18.0%
	Parental disability	Did your female/male guardian have a long time being sick on bed when you were young? (yes[a] or no)	22.9%
		Did your female/male guardian have a serious deformity when you were young? (yes[a] or no)	

Abbreviation: ACEs: adverse childhood experiences.

[a] Answers indicate thresholds for ACEs.

[b] Calculated based on dates of birth and their parental death.

[c] Calculated based on dates of birth and their sibling's death.

© 2021 Lin L et al. *JAMA Network Open*.

eTable 2. Association Between the Number of Conventional ACEs and Subsequent Chronic Diseases and Multimorbidity in Adulthood.

	**OR (95% CI) by No. of ACEs**					***P* for trend**
	**0**	**1**	**2**	**3**	**≥4**	
**Model 1**[a]						
**Chronic diseases**						
Hypertension	1 [Reference][c]	1.05 [0.96, 1.15]	0.98 [0.88, 1.10]	0.94 [0.81, 1.10]	0.97 [0.74, 1.26]	.45
Dyslipidemia	1 [Reference]	1.05 [0.92, 1.19]	1.05 [0.89, 1.23]	1.08 [0.86, 1.35]	**1.43 [1.00, 2.03]**[d]	.13
Diabetes	1 [Reference]	0.96 [0.85, 1.09]	1.08 [0.93, 1.24]	1.12 [0.92, 1.36]	1.06 [0.75, 1.51]	.18
Heart disease	1 [Reference]	0.90 [0.79, 1.03]	0.97 [0.83, 1.13]	1.21 [0.98, 1.48]	1.25 [0.89, 1.78]	.13
Stroke	1 [Reference]	**1.35 [1.01, 1.80]**	1.19 [0.84, 1.70]	1.40 [0.88, 2.22]	**2.02 [1.03, 3.95]**	.04
Chronic lung disease	1 [Reference]	1.08 [0.93, 1.25]	**1.38 [1.17, 1.64]**	**1.67 [1.33, 2.08]**	**2.17 [1.52, 3.09]**	<.001
Asthma	1 [Reference]	1.17 [0.93, 1.46]	**1.36 [1.05, 1.76]**	**1.57 [1.12, 2.21]**	**2.65 [1.65, 4.25]**	<.001
Liver disease	1 [Reference]	0.92 [0.75, 1.14]	1.09 [0.85, 1.39]	1.31 [0.95, 1.81]	1.15 [0.64, 2.05]	.12
Cancer	1 [Reference]	0.85 [0.57, 1.27]	0.82 [0.50, 1.34]	**1.80 [1.07, 3.04]**	0.61 [0.15, 2.51]	.46
Digestive disease	1 [Reference]	**1.18 [1.06, 1.32]**	**1.63 [1.43, 1.85]**	**1.99 [1.68, 2.37]**	**2.87 [2.16, 3.83]**	<.001
Kidney disease	1 [Reference]	1.01 [0.85, 1.20]	**1.32 [1.09, 1.61]**	**1.33 [1.02, 1.75]**	**2.07 [1.39, 3.07]**	<.001
Arthritis	1 [Reference]	**1.17 [1.06, 1.29]**	**1.50 [1.33, 1.69]**	**1.73 [1.46, 2.04]**	**2.08 [1.57, 2.78]**	<.001
Psychiatric disease	1 [Reference]	0.92 [0.62, 1.37]	1.22 [0.79, 1.90]	**2.30 [1.41, 3.76]**	**2.99 [1.45, 6.16]**	<.001
Memory-related disease	1 [Reference]	0.96 [0.69, 1.34]	**1.48 [1.04, 2.10]**	1.51 [0.94, 2.43]	1.60 [0.73, 3.51]	.009
**Multimorbidity**	1 [Reference]	1.09 [0.99, 1.20]	**1.43 [1.27, 1.61]**	**1.50 [1.26, 1.78]**	**2.52 [1.81, 3.50]**	<.001

© 2021 Lin L et al. *JAMA Network Open*.

eTable 2. Continued.

	OR (95% CI) by No. of ACEs					*P* for trend
	0	1	2	3	≥4	
**Model 2**[b]						
**Chronic disease**						
Hypertension	1 [Reference]	1.05 [0.94,1.17]	1.01 [0.88,1.16]	0.99 [0.82,1.20]	0.85 [0.60,1.20]	.66
Dyslipidemia	1 [Reference]	1.09 [0.93,1.28]	1.15 [0.95,1.39]	**1.34 [1.03,1.73]**	**1.71 [1.11,2.62]**	.003
Diabetes	1 [Reference]	1.01 [0.87,1.17]	1.12 [0.94,1.34]	1.20 [0.94,1.52]	0.97 [0.61,1.53]	.15
Heart disease	1 [Reference]	0.99 [0.85,1.15]	1.04 [0.86,1.26]	**1.39 [1.09,1.77]**	**1.52 [1.00,2.31]**	.009
Stroke	1 [Reference]	1.32 [0.93,1.88]	1.00 [0.64,1.57]	1.41 [0.81,2.45]	**3.07 [1.51,6.23]**	.048
Chronic lung disease	1 [Reference]	1.02 [0.85,1.21]	**1.31 [1.07,1.61]**	**1.37 [1.04,1.80]**	**2.24 [1.48,3.40]**	<.001
Asthma	1 [Reference]	1.10 [0.84,1.44]	1.35 [0.99,1.84]	**1.66 [1.12,2.46]**	**2.87 [1.64,5.02]**	<.001
Liver disease	1 [Reference]	0.84 [0.66,1.08]	1.05 [0.79,1.40]	1.21 [0.83,1.77]	0.99 [0.49,2.00]	.41
Cancer	1 [Reference]	0.91 [0.57,1.47]	0.91 [0.50,1.64]	1.80 [0.94,3.45]	0.86 [0.20,3.67]	.40
Digestive disease	1 [Reference]	1.12 [0.98,1.28]	**1.65 [1.42,1.93]**	**1.96 [1.59,2.41]**	**3.13 [2.22,4.43]**	<.001
Kidney disease	1 [Reference]	0.88 [0.72,1.09]	1.15 [0.91,1.46]	1.29 [0.94,1.77]	**1.93 [1.20,3.09]**	.005
Arthritis	1 [Reference]	**1.18 [1.05,1.33]**	**1.44 [1.25,1.67]**	**1.73 [1.41,2.11]**	**2.26 [1.59,3.20]**	<.001
Psychiatric disease	1 [Reference]	0.90 [0.57,1.44]	1.41 [0.85,2.33]	**2.30 [1.29,4.11]**	**3.57 [1.61,7.93]**	<.001
Memory-related disease	1 [Reference]	1.00 [0.66,1.51]	**1.68 [1.09,2.60]**	1.55 [0.86,2.80]	2.10 [0.86,5.10]	.008
**Multimorbidity**	1 [Reference]	1.06 [0.94,1.20]	**1.45 [1.25,1.68]**	**1.51 [1.22,1.86]**	**2.79 [1.85,4.22]**	<.001

Abbreviations: OR: odds ratio; ACEs: adverse childhood experiences.

[a] Model 1 was the crude model.

[b] Model 2 was adjusted for age, sex, ethnicity, marital status, educational level, rural or urban residence, smoking and drinking status, annual per capita household expenditure level, and childhood economic hardship.

[c] Reference: No ACE exposure.

[d] Bold OR [95% CI] indicates statistical significance.

© 2021 Lin L et al. *JAMA Network Open*.

eTable 3. Association Between the Number of ACEs and Subsequent Chronic Diseases in Adulthood, With Imputed Data Sets.

	OR (95% CI) by No. of ACEs					*P* for trend
**Chronic disease**	0	1	2	3	≥4	
**Model 1**[a]						
Hypertension	1 [Reference][c]	1.03 [0.92, 1.15]	1.08 [0.97, 1.21]	1.02 [0.91, 1.15]	0.99 [0.88, 1.11]	.87
Dyslipidemia	1 [Reference]	1.02 [0.88, 1.20]	1.07 [0.92, 1.24]	1.06 [0.89, 1.25]	1.06 [0.89, 1.26]	.46
Diabetes	1 [Reference]	0.99 [0.85, 1.14]	1.04 [0.90, 1.20]	1.02 [0.87, 1.20]	1.10 [0.94, 1.29]	.19
Heart disease	1 [Reference]	0.99 [0.85, 1.15]	1.06 [0.91, 1.22]	1.13 [0.96, 1.34]	1.08 [0.92, 1.27]	.12
Stroke	1 [Reference]	0.96 [0.68, 1.35]	1.31 [0.95, 1.80]	**1.61 [1.12, 2.30]**[d]	**1.67 [1.16, 2.39]**	<.001
Chronic lung disease	1 [Reference]	1.10 [0.90, 1.33]	**1.48 [1.22, 1.78]**	**1.91 [1.59, 2.29]**	**2.41 [2.00, 2.90]**	<.001
Asthma	1 [Reference]	1.09 [0.83, 1.43]	**1.46 [1.11, 1.91]**	**1.88 [1.39, 2.54]**	**2.32 [1.77, 3.05]**	<.001
Liver disease	1 [Reference]	0.98 [0.75, 1.27]	**1.29 [1.00, 1.67]**	1.26 [0.95, 1.68]	**1.56 [1.20, 2.03]**	<.001
Cancer	1 [Reference]	1.04 [0.65, 1.66]	1.17 [0.73, 1.89]	0.87 [0.48, 1.57]	1.25 [0.74, 2.10]	.56
Digestive disease	1 [Reference]	**1.30 [1.14, 1.48]**	**1.57 [1.38, 1.80]**	**1.91 [1.63, 2.23]**	**2.46 [2.15, 2.83]**	<.001
Kidney disease	1 [Reference]	**1.23 [1.00, 1.51]**	**1.40 [1.13, 1.74]**	**1.61 [1.28, 2.02]**	**2.03 [1.61, 2.57]**	<.001
Arthritis	1 [Reference]	**1.16 [1.03, 1.31]**	**1.51 [1.34, 1.71]**	**1.79 [1.57, 2.04]**	**2.26 [1.97, 2.59]**	<.001
Psychiatric disease	1 [Reference]	0.95 [0.62, 1.46]	1.28 [0.82, 2.00]	1.48 [0.93, 2.36]	**2.27 [1.44, 3.58]**	<.001
Memory-related disease	1 [Reference]	1.18 [0.81, 1.71]	**1.48 [1.01, 2.16]**	**1.57 [1.03, 2.39]**	**2.11 [1.40, 3.17]**	<.001

© 2021 Lin L et al. *JAMA Network Open*.

eTable 3. Continued.

	OR (95% CI) by No. of ACEs					*P* for trend
**Chronic disease**	0	1	2	3	⩾4	
**Model 2**[b]						
Hypertension	1 [Reference]	0.99 [0.89, 1.11]	1.02 [0.90, 1.14]	0.93 [0.82, 1.05]	0.95 [0.86, 1.06]	.39
Dyslipidemia	1 [Reference]	1.06 [0.90, 1.24]	1.14 [0.98, 1.33]	1.15 [0.96, 1.37]	**1.20 [1.01, 1.44]**	.03
Diabetes	1 [Reference]	0.99 [0.85, 1.14]	1.05 [0.91, 1.22]	1.03 [0.87, 1.21]	1.11 [0.94, 1.31]	.16
Heart disease	1 [Reference]	1.01 [0.87, 1.17]	1.09 [0.94, 1.27]	**1.20 [1.01, 1.42]**	1.15 [0.98, 1.36]	.02
Stroke	1 [Reference]	0.91 [0.65, 1.29]	1.21 [0.87, 1.68]	1.43 [0.98, 2.07]	1.41 [0.96, 2.08]	.009
Chronic lung disease	1 [Reference]	1.04 [0.86, 1.27]	**1.35 [1.11, 1.63]**	**1.67 [1.38, 2.02]**	**2.02 [1.66, 2.45]**	<.001
Asthma	1 [Reference]	1.04 [0.79, 1.37]	**1.33 [1.00, 1.75]**	**1.64 [1.20, 2.23]**	**1.94 [1.45, 2.59]**	<.001
Liver disease	1 [Reference]	0.99 [0.76, 1.28]	**1.30 [1.00, 1.67]**	1.27 [0.95, 1.70]	**1.58 [1.20, 2.07]**	<.001
Cancer	1 [Reference]	1.07 [0.67, 1.72]	1.25 [0.78, 2.02]	0.94 [0.51, 1.73]	1.34 [0.77, 2.33]	.39
Digestive disease	1 [Reference]	**1.30 [1.14, 1.49]**	**1.58 [1.37, 1.81]**	**1.91 [1.63, 2.25]**	**2.38 [2.06, 2.75]**	<.001
Kidney disease	1 [Reference]	1.20 [0.97, 1.47]	**1.32 [1.07, 1.64]**	**1.47 [1.16, 1.86]**	**1.81 [1.42, 2.31]**	<.001
Arthritis	1 [Reference]	**1.14 [1.01, 1.29]**	**1.47 [1.29, 1.66]**	**1.70 [1.48, 1.96]**	**2.03 [1.75, 2.34]**	<.001
Psychiatric disease	1 [Reference]	0.95 [0.62, 1.45]	1.28 [0.82, 1.99]	1.47 [0.91, 2.35]	**2.15 [1.34, 3.43]**	<.001
Memory-related disease	1 [Reference]	1.13 [0.78, 1.64]	1.36 [0.93, 2.01]	1.39 [0.90, 2.16]	**1.80 [1.17, 2.76]**	.005

Abbreviations: OR: odds ratio; ACEs: adverse childhood experiences.

[a] Model 1 was the crude model.

[b] Model 2 was adjusted for age, sex, ethnicity, marital status, educational level, rural or urban residence, smoking and drinking status, annual per capita household expenditure level, and childhood economic hardship.

[c] Reference: No ACE exposure.

[d] Bold OR [95% CI] indicates statistical significance.

© 2021 Lin L et al. *JAMA Network Open*.

eTable 4. Association Between the Number of ACEs and Multimorbidity in the Overall Study Population and Subgroups, With Imputed Data Sets.

	OR (95% CI) by No. of ACEs[a]					*P* for trend	*P* for interaction
**Characteristic**	0	1	2	3	≥4		
**Overall study population**	1 [Reference][b]	**1.13 [1.00, 1.26]**[c]	**1.47 [1.30, 1.66]**	**1.67 [1.44, 1.94]**	**1.96 [1.72, 2.25]**	<.001	
**Subgroups**							
**Age, y**							.06
<60	1 [Reference]	1.13 [0.97, 1.31]	**1.52 [1.27, 1.81]**	**1.72 [1.42, 2.09]**	**2.06 [1.70, 2.50]**	<.001	
≥60	1 [Reference]	1.12 [0.95, 1.32]	**1.44 [1.22, 1.71]**	**1.63 [1.34, 1.99]**	**1.91 [1.59, 2.28]**	<.001	
**Sex**							.30
Male	1 [Reference]	1.08 [0.91, 1.27]	**1.43 [1.19, 1.71]**	**1.53 [1.25, 1.87]**	**1.86 [1.53, 2.26]**	<.001	
Female	1 [Reference]	**1.16 [1.00, 1.35]**	**1.51 [1.30, 1.76]**	**1.82 [1.50, 2.21]**	**2.07 [1.74, 2.47]**	<.001	
**Childhood economic hardship**							.92
Yes	1 [Reference]	1.13 [0.90, 1.41]	**1.46 [1.17, 1.83]**	**1.71 [1.35, 2.18]**	**1.96 [1.58, 2.42]**	<.001	
No	1 [Reference]	1.12 [0.98, 1.29]	**1.48 [1.27, 1.71]**	**1.63 [1.36, 1.96]**	**1.99 [1.66, 2.38]**	<.001	
**Educational level completed**							.75
Primary school or below	1 [Reference]	**1.17 [1.00, 1.36]**	**1.54 [1.32, 1.80]**	**1.72 [1.45, 2.06]**	**2.04 [1.73, 2.40]**	<.001	
Middle school or above	1 [Reference]	1.06 [0.89, 1.25]	**1.35 [1.12, 1.62]**	**1.57 [1.24, 2.00]**	**1.78 [1.42, 2.23]**	<.001	
**Annual per capita household expenditure level**							.96
Tertile 1	1 [Reference]	1.20 [0.99, 1.45]	**1.43 [1.16, 1.76]**	**1.66 [1.30, 2.11]**	**2.10 [1.66, 2.66]**	<.001	
Tertile 2	1 [Reference]	1.13 [0.92, 1.37]	**1.58 [1.29, 1.94]**	**1.76 [1.37, 2.27]**	**1.93 [1.55, 2.41]**	<.001	
Tertile 3	1 [Reference]	1.05 [0.86, 1.28]	**1.41 [1.15, 1.74]**	**1.61 [1.25, 2.08]**	**1.85 [1.46, 2.36]**	<.001	

Abbreviations: OR: odds ratio; ACEs: adverse childhood experiences.

[a] The model was adjusted for age, sex, ethnicity, marital status, educational level, rural or urban residence, smoking and drinking status, annual per capita household expenditure level, and childhood economic hardship, except for the stratified variables in each subgroup.

[b] Reference: No ACE exposure.

[c] Bold OR [95% CI] indicates statistical significance.

© 2021 Lin L et al. *JAMA Network Open*.

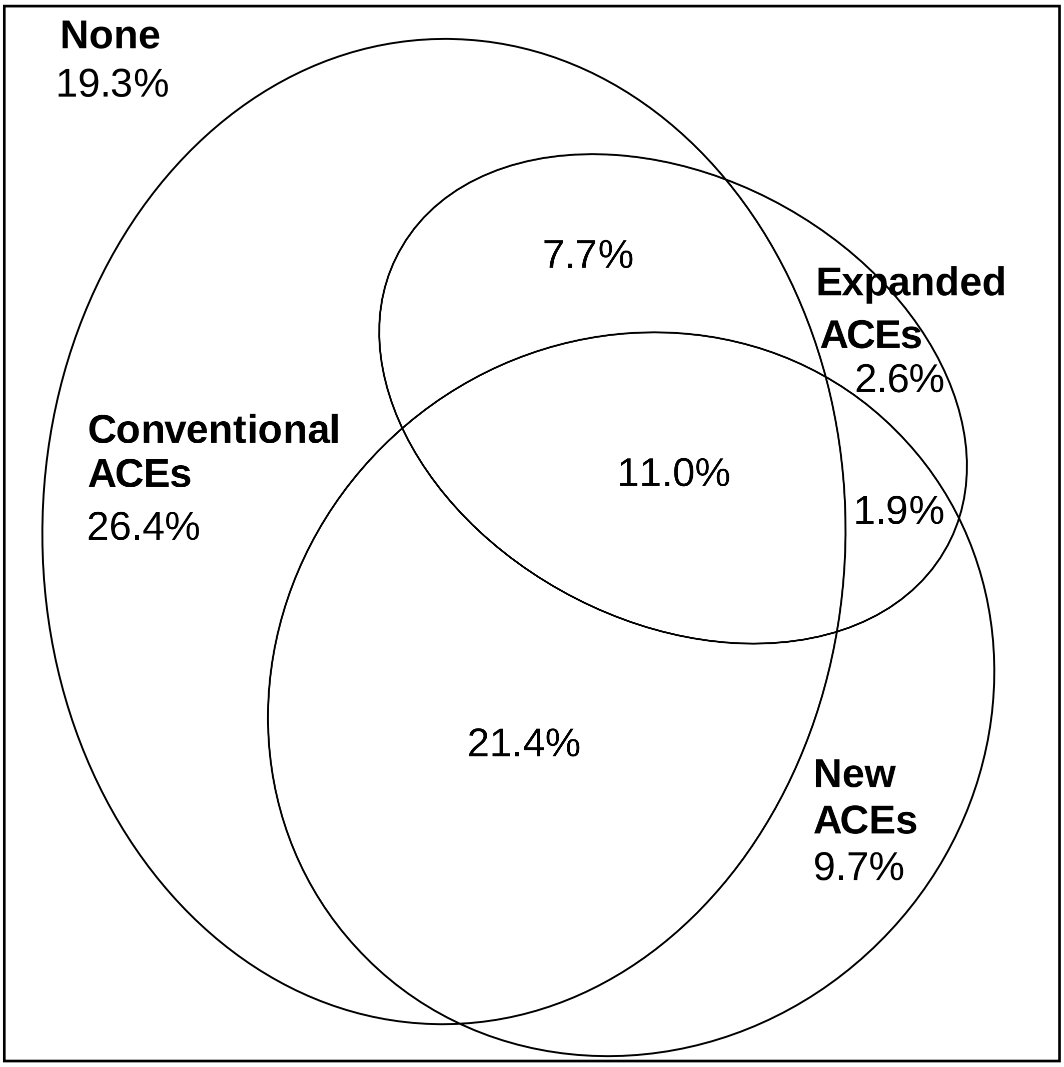

.

eFigure 1. Overlapping Exposure to Conventional, Expanded, and New ACEs. Conventional ACEs included physical abuse, emotional neglect, household substance abuse, household mental illness, domestic violence, incarcerated household member, and parental separation or divorce. Expanded ACEs included unsafe neighborhood and bullying. New ACEs included parental death, sibling death, and parental disability.

© 2021 Lin L et al. *JAMA Network Open*.

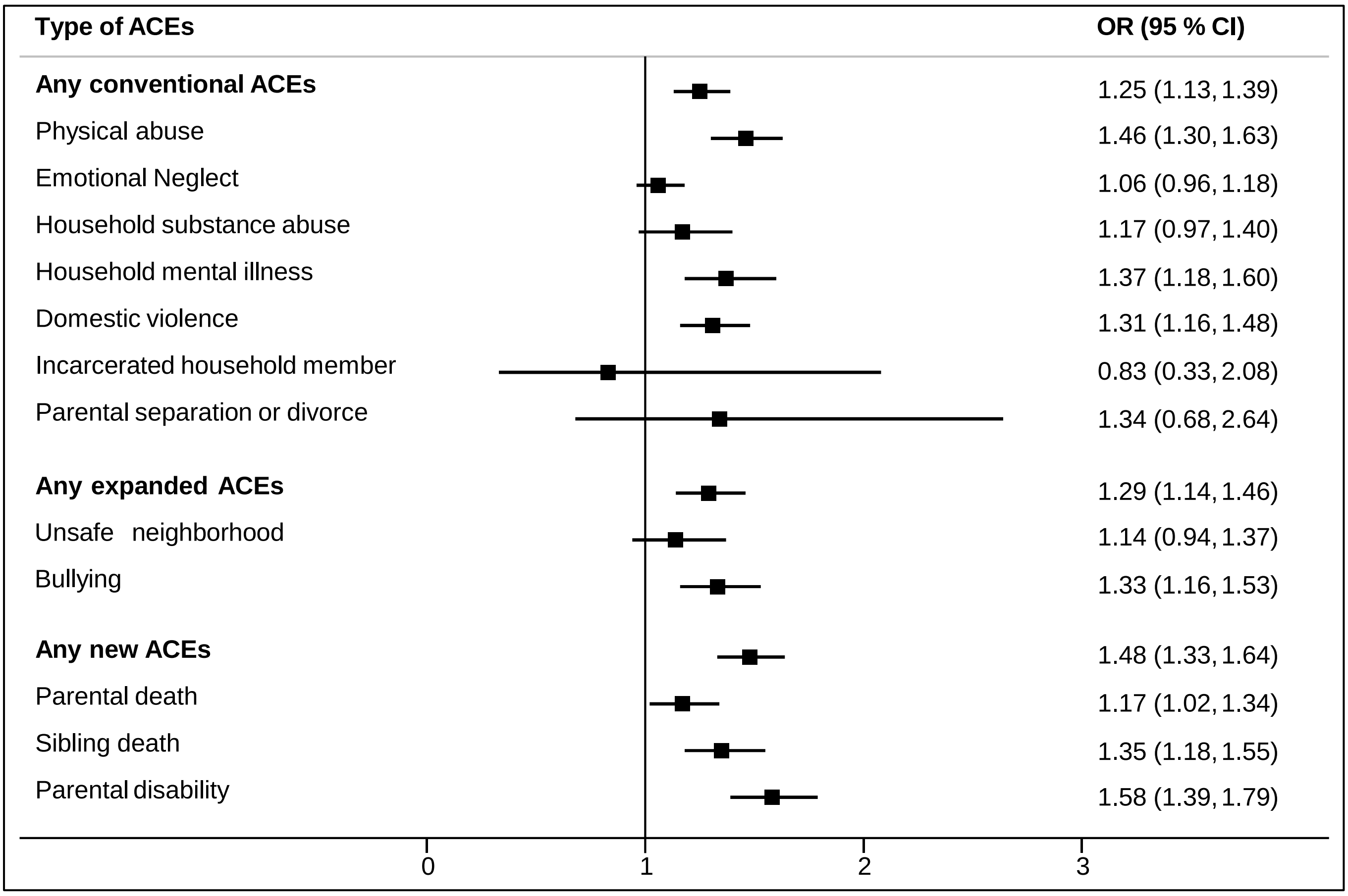

eFigure 2. Association Between Individual ACE Indicator and Multimorbidity. Model was adjusted for age, sex, ethnicity, marital status, educational level, rural or urban residence, smoking and drinking status, annual per capita household expenditure level, and childhood economic hardship.

© 2021 Lin L et al. *JAMA Network Open*.

References

1. Zhao Y, Hu Y, Smith JP, Strauss J, Yang G. Cohort profile: the China Health and Retirement Longitudinal Study (CHARLS). *Int J Epidemiol*. Feb 2014;43(1):61-8. doi:10.1093/ije/dys203

2. Chobanian AV, Bakris GL, Black HR, et al. The Seventh Report of the Joint National Committee on Prevention, Detection, Evaluation, and Treatment of High Blood Pressure - The JNC 7 Report. *Jama-Journal of the American Medical Association*. May 2003;289(19):2560-2572. doi:10.1001/jama.289.19.2560

3. Standards of medical care in diabetes--2010. *Diabetes care*. Jan 2010;33 Suppl 1(Suppl 1):S11-61. doi:10.2337/dc10-S011

4. Johnston MC, Crilly M, Black C, Prescott GJ, Mercer SW. Defining and measuring multimorbidity: a systematic review of systematic reviews. *European journal of public health*. Feb 1 2019;29(1):182-189. doi:10.1093/eurpub/cky098

5. NBSC. National Bureau of Statistics of China. Administrative area codes and urban-rural codes for statistics in 2014 (up to October 31, 2014). http://www.stats.gov.cn/tjsj/tjbz/tjyqhdmhcxhfdm/2014/index.html (Accessed May 24, 2020).

6. Angus D, Salman Z. Guidelines for Constructing Consumption Aggregates for Welfare Analysis. *LSMS Working Paper;No 135 World Bank*. 2002;© World Bank. https://openknowledge.worldbank.org/handle/10986/14101 License: CC BY 3.0 IGO.

© 2021 Lin L et al. *JAMA Network Open*.
